# Bioenergetic profiles of peripheral mononuclear cells and systemic inflammation in women with Interstitial Cystitis/Bladder Pain Syndrome (IC/BPS)

**DOI:** 10.1371/journal.pone.0298981

**Published:** 2024-02-15

**Authors:** Parveen Kumar, Robert A. Oster, Dean G. Assimos, Timothy J. Ness, Tanecia Mitchell

**Affiliations:** 1 Department of Urology, University of Alabama at Birmingham, Birmingham, AL, United States of America; 2 Department of Medicine, University of Alabama at Birmingham, Birmingham, AL, United States of America; 3 Department of Anesthesiology, University of Alabama at Birmingham, Birmingham, AL, United States of America; University of Illinois, UNITED STATES

## Abstract

Inflammation is thought to contribute to the etiology of interstitial cystitis/bladder pain syndrome (IC/BPS). It is well-known that disruption in metabolism in immune cells contributes to inflammation in several inflammatory diseases. The purpose of this study was to investigate whether cellular bioenergetics is altered in monocytes and lymphocytes from women with IC/BPS, and if these alterations correlate with systemic inflammatory markers. Age and BMI matched adult healthy women (HS; n = 18) and women with IC/BPS (n = 18) were included in the study. Blood was collected to assess cellular bioenergetics in monocytes and lymphocytes using a Seahorse XF96 Analyzer and plasma cytokine levels were measured using Meso Scale Discovery immunoassays. The correlation between bioenergetic parameters, cytokines, and demographics was determined using Pearson correlation coefficients. Means of the two groups were compared using the two-group t-test. Patients with IC/BPS had reduced monocyte oxygen consumption rates and glycolytic rates compared to healthy subjects. In contrast, lymphocytes from these patients had increased oxygen consumption rates and glycolytic rates. Several cytokines and chemokines including Interferon-gamma (IFN-ɣ), tumor necrosis factor alpha (TNF-ɑ), Interleukin-6 (IL-6), Interleukin-8 (IL-8) and vascular endothelial growth factor (VEGF) levels were significantly elevated in the plasma of patients with IC/BPS. However, Transforming growth factor (TGF-β) and Interleukin-10 (IL-10) levels were significantly decreased in IC/BPS patients compared to HS. In addition, Interferon gamma (IFN-ɣ), TNF-ɑ, IL-8, and TGF-β levels correlated with several bioenergetic parameters in monocytes or lymphocytes from healthy subjects. In contrast, TNF-ɑ and IL-8 correlated with bioenergetic parameters in monocytes from IC/BPS patients. Monocyte and lymphocyte cellular bioenergetics and plasma cytokine levels are different in patients with IC/PBS compared to HS. It appears that systemic inflammation is greater in this cohort which may negatively impact immune cell function. The relationship between cellular bioenergetics and inflammation in monocytes and lymphocytes could be important in understanding the pathogenesis of IC/PBS and warrants further investigation.

## Introduction

Interstitial cystitis/bladder pain syndrome (IC/BPS) is a condition that occurs primarily in women and consists of symptomatic pelvic and bladder pain for more than six months [1]. Approximately 3.3 to 7.9 million women are affected by IC/BPS in the United States [2]. Patients with IC/BPS often experience the need to urinate urgently and frequently. The optimal way to diagnose IC/BPS is limited and is based on the exclusion of other urological/gynecological disorders such as urinary tract infections, overactive bladder, or pelvic inflammatory disease. The exact cause of IC/BPS appears to multifactorial and is not well understood. Some factors thought to contribute to the condition include genetics, autoimmune diseases, and infection [3]. In addition, patients with IC/BPS have a higher risk of developing additional co-morbidities such as fibromyalgia, endometriosis, and irritable bowel disease [4–6]. Thus, it appears the pathophysiology of IC/BPS could be largely influenced by systemic factors.

Inflammation has been suggested to contribute to IC/BPS and to be associated with pain in afflicted patients [1, 7]. During chronic inflammation, the bladder is infiltrated with mononuclear cells such as macrophages, lymphocytes, eosinophils and mast cells [1]. These cells elicit an immune response within the bladder and recruit other inflammatory cells from the circulation. Pro-inflammatory cytokines and chemokines, such as interleukin-6 (IL-6) and tumor necrosis factor (TNF-ɑ), are associated with mast cell activation and are elevated in the serum of this cohort [8]. TNF-ɑ is known to play a role in apoptosis and immune system development [9]. It is stimulated by the pro-inflammatory cytokine, interferon-gamma (IFN-ɣ) [10]. Such an environment in the circulation could promote a pro-inflammatory response and negatively affect immune cell function.

A number of diseases such as diabetes, atherosclerosis, Alzheimer’s disease, chronic kidney disease, and cancer have been associated with systemic oxidative stress and mitochondrial dysfunction [11]. Mitochondria play a critical role in immune cell function and their ability to respond to immune activation [12]. Several studies have evaluated metabolism in peripheral blood mononuclear cells isolated from those with a variety of conditions including non-alcoholic fatty liver disease [13], mild cognitive impairment [14], Parkinson’s disease [15], fibromyalgia [16], and human immunodeficiency virus [17]. We have previously determined that monocytes have decreased cellular bioenergetics in patients with calcium oxalate kidney stone disease compared to age and gender matched healthy subjects [18]. A noticeable similarity among the aforementioned disease processes is their association with inflammation and other systemic disorders.

The ability to isolate circulating immune cells to assess disease progression or treatment is an area of research that has many potential translational implications. The objective of this study was to determine whether circulating immune cells and inflammatory markers may be affected in IC/BPS. We assessed bioenergetic profiles in circulating monocytes and lymphocytes and plasma cytokine and chemokine levels from women with IC/BPS and age-matched healthy women. Monocytes and lymphocytes are derived from myeloid and lymphoid lineage, respectively, and are involved in innate and adaptive immunity. Our results suggest that monocytes and lymphocytes may contribute to the pathogenesis of IC/BPS and that inflammatory cytokines may be linked. These findings could aid in the development of novel therapeutic avenues for these patients to improve clinical outcomes, and methods of monitoring treatment responses. To our knowledge, this is the first study to assess immunometabolism in a cohort of patients with IC/BPS.

## Materials and methods

### Study participants

This study was approved by the University of Alabama at Birmingham Institutional Review Board. Informed written consent was obtained from all study participants prior to beginning the study. Age and BMI matched healthy women were recruited from the Birmingham, AL area and denied any urological disorders or symptoms. Women were only studied since IC/BPS primarily occurs in females and the ability to recruit men with this condition was limited. Detailed clinical characteristics of patients with IC/BPS were abstracted from electronic medical records. All IC/BPS subjects reported abdominal/pelvic pain, pressure, or discomfort associated with either bladder filling or frequent emptying, and did not have signs or symptoms which met the exclusionary criteria such as an active urinary tract infection. These symptoms met the IC/BPS definition generated by the Society of Urodynamics and Female Urology which continues to be endorsed by the American Urological Association’s IC/BPS guidelines panel [19].

### Sample collection and cell isolation

Blood samples were collected from patients with IC/BPS prior to receiving pain management treatment at the UAB Medical Center between 7/1/2014 to 10/1/2016. Healthy subjects (HS) had their blood drawn at the UAB Center for Clinical and Translational Science Clinical Research Unit. All blood (15–25 mL) was centrifuged to collect the buffy coat/red blood cell layers [20]. The cells were diluted with RPMI media 1640 (Life Technologies, Grand Island, NY) and separated by Ficoll-paque density gradient (Sigma Aldrich, St. Louis, MO) to collect the mononuclear cell layer. Magnetic bead antibodies (anti-CD14, anti-CD65, and anti-CD235a) were used to isolate monocytes and lymphocytes as recommended by Miltenyi Biotec Inc. (San Diego, CA). Cell count was determined using the Bio-Rad TC20 Automated Cell Counter (Bio-Rad, Hercules, CA).

### Cellular bioenergetic functional assays and analyses

The Seahorse XFe96 Analyzer (Agilent Technologies, Santa Clara, CA) was used to assess cellular bioenergetics. This technology assesses mitochondrial respiration and glycolysis simultaneously in cells in real-time. Monocytes and lymphocytes were plated (150,000 cells/well) on Cell-Tak coated Seahorse XF96 plates. After establishing baseline readings, the “Mitochondrial Stress Test” (MST) was initiated by injecting oligomycin (0.5 μg/mL), FCCP (0.6 μM), and antimycin A (10 μM) over time into the cellular media. The oxygen consumption rates (OCR) generated from the MST assay was used to determine several parameters including basal respiration, ATP-linked respiration, Proton Leak, Maximal respiration, Reserve Capacity, and Non-mitochondrial respiration. Basal respiration is representative of the energetic demand of cells at baseline levels. ATP-linked OCR represents the respiration used to produce ATP from the mitochondria. Proton leak is the respiration not coupled to ATP generation. Maximal OCR represents the highest level of respiration cells can generate. Reserve capacity is the ability of the cells to respond to an energetic demand. Non-mitochondrial respiration is indicative of cellular respiration from other sources outside of the mitochondria. In addition, we assessed glycolysis by determining the flux of protons in the cellular media and calculated the basal extracellular acidification rates (ECAR) and oligo-sensitive ECAR. All data were analyzed using the Wave software (Santa Clara, CA). All OCR and ECAR were normalized to cell count. Individual mitochondrial and glycolytic parameters were determined as previously described [18].

### Multiplex immunoassay plasma analysis

Plasma was collected from study participants and frozen at -80°C until evaluation. IFN-ɣ, IL-1β, IL-2, IL-4, IL-6, IL-8, IL-10, IL-12p70, IL-13, and TNF-ɑ analyte levels were measured using a human V-Plex Plus Pro-Inflammatory 10-plex Panel; GM-CSF, IL-12/IL-23P40, IL-15, IL-16, IL-17A, IL-1ɑ, IL-5, IL-7, TNF-β, and VEGF-A analyte levels were measured using a human V-Plex Plus Cytokine Panel (Meso Scale Discovery-MSD, Gaithersburg, MD). All samples were diluted 2-fold in assay diluent according to the manufacturer’s instructions. Samples were analyzed on a multi-array plate by electrochemiluminescence using the Sector Imager 2400 (Meso Scale Discovery-MSD, Gaithersburg, MD). Data were analyzed using the MSD Workbench software.

### Statistical analysis

Descriptive statistics, including means and standard deviations for continuous variables and frequencies and proportions for categorical variables, were calculated. Comparisons of means between the two groups of interest (HS vs IC/BPS) were performed using the two-group t-test, or the adjusted (Satterthwaite) t-test as needed. Correlation analyses were performed using Pearson correlation coefficients; these coefficients were tested for statistical significance. Distributions of continuous variables were examined for normality using stem-and-leaf plots, box plots, normal probability plots, and the Kolmogorov-Smirnov test, and it was determined that these variables follow at least an approximate normal distribution. Statistical tests were two-sided and were performed using a 5% significance level (i.e., α = 0.05). Statistical analyses were performed using SAS software (version 9.4; SAS Institute, Inc., Cary, NC).

## Results

### Clinical demographics and parameters

Healthy women (n = 18) and those diagnosed with IC/BPS (n = 18) were enrolled in the study (**Table 1**). The mean ages were 43.9 ± 10.3 years and 49.6 ± 15.3 years in the HS and IC/BPS cohort respectively, which were not significantly different (p = 0.202). The body mass index (BMI) was similar (p = 0.575) among the two groups (28.8 ± 7.0 kg/m^2^ HS vs. 30.3 ± 8.2 kg/m^2^ IC/BPS). Of the patients with IC/BPS, sixteen (88.9%) had a non-ulcerative endoscopic diagnosis and two (11.1%) had an ulcerative endoscopic diagnosis. Some patients also had active or a history of hypertension (7; 38.8%), hypothyroidism (4; 22.2%), fibromyalgia (2; 11.1%), or endometriosis (1; 5.5%) at the time of the blood draw. These co-morbidities and phenotypic differences did not impact responses subsequently profiled.

**Table 1:** Demographics and characteristics of study cohort.

** **	**HS**	**IC/BPS**
**Demographics**		
Number	18	18
Age, years	43.9 ± 10.3	49.6 ± 15.3
BMI, kg/m^2^	28.8 ± 7.0	30.3 ± 8.2
**Diagnosis** *		
Ulcerative	—	2/18 (11.1%)
Non-ulcerative	—	16/18 (88.9%)
**Comorbidities**		
Hypertension	—	7/18 (38.8%)
Hypothyroidism	—	4/18 (22.2%)
Fibromyalgia	—	2/18 (11.1%)
Endometriosis	—	1/18 (5.5%)

—Values are expressed in Mean ± SD.

—HS = Healthy Subjects; IC/BPS = Interstitial cystitis/bladder pain syndrome

—BMI = body mass index.

—IC/PBS compared to HS. There are no statistically significant group differences for age (p = 0.202) or BMI (p = 0.575).

*Ulcerative indicates Hunner’s patch observed on cystoscopy.

(—), not applicable.

### Monocyte mitochondrial respiration and glycolysis

**Fig 1:**
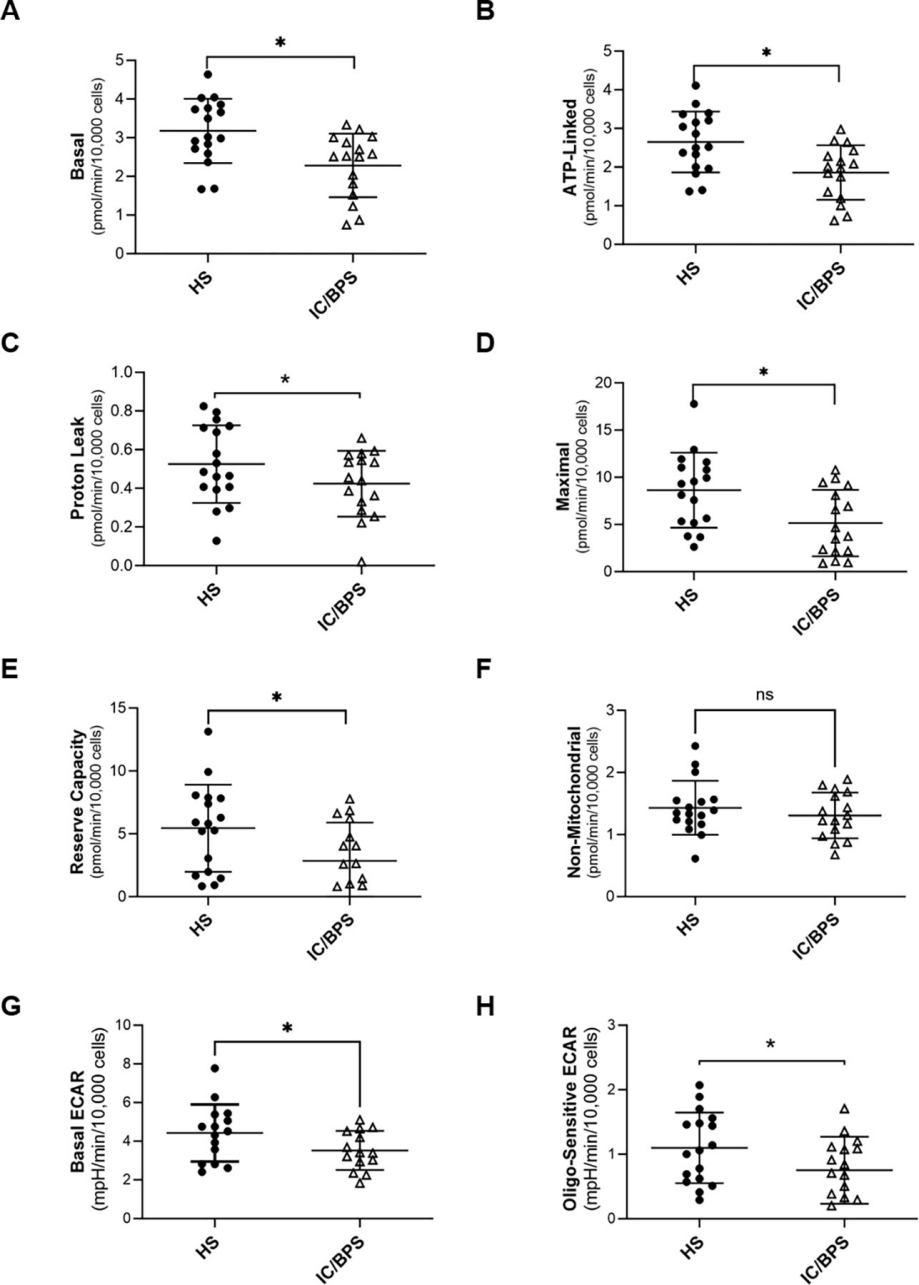
Monocyte mitochondrial respiration and glycolysis in healthy subjects and patients with Interstitial Cystitis/Bladder Pain Syndrome (IC/BPS). Distribution of (A) basal, (B) ATP-linked, (C) Proton Leak, (D) Maximal, (E) Reserve Capacity, and (F) Non-mitochondrial OCR parameters as well as (G) Basal ECAR and (H) Oligo-sensitive ECAR parameters in monocytes from study participants. Data expressed as mean ± SD, n = 5–6 replicates; up to n  = 17 HS, n = 16 IC/BPS. *p<0.05, different from HS.

**Fig 1** shows the distribution of basal, ATP-linked, proton leak, maximal, reserve capacity, and non-mitochondrial OCR in monocytes from HS and IC/BPS patients. Basal respiration was significantly decreased in IC/BPS compared to HS (3.18 ± 0.83 vs 2.28 ± 0.82 pmol/min/10,000 cells; p = 0.004) (**Fig 1A**). Consistent with the basal OCR responses, ATP-linked OCR (2.65 ± 0.78 vs 1.86 ± 0.71 pmol/min/10,000 cells; p = 0.005) (**Fig 1B**), Proton leak (0.55 ± 0.18 vs 0.41 ± 0.16 pmol/min/10,000 cells; p = 0.014) (**Fig 1C**), maximal respiration (8.63 ± 3.98 vs 5.14 ± 3.51 pmol/min/10,000 cells; p = 0.012) (**Fig 1D**), and reserve capacity (5.45 ± 3.46 vs 2.86 ± 3.04 pmol/min/10,000 cells; p = 0.030) (**Fig 1E**) were all significantly reduced in patients with IC/BPS compared to HS. However, non-mitochondrial respiration was not significantly different between the two cohorts (1.43 ± 0.43 vs 1.31 ± 0.37 pmol/min/10,000 cells; p = 0.388) (**Fig 1F**). The XF analyzer also provided extracellular acidification rate (ECAR) data shown in **Fig 1G and 1H.** Basal ECAR (4.43 ± 1.48 vs 3.52 ± 1.00 mpH/min/10,000 cells; p = 0.033) and Oligo-sensitive ECAR (1.15 ± 0.52 vs 0.76 ± 0.38 mpH/min/10,000 cells; p = 0.030) were significantly decreased in patients with IC/BPS compared to HS (**Fig 1G and 1H**).

### Lymphocyte mitochondrial respiration and glycolysis

**Fig 2:**
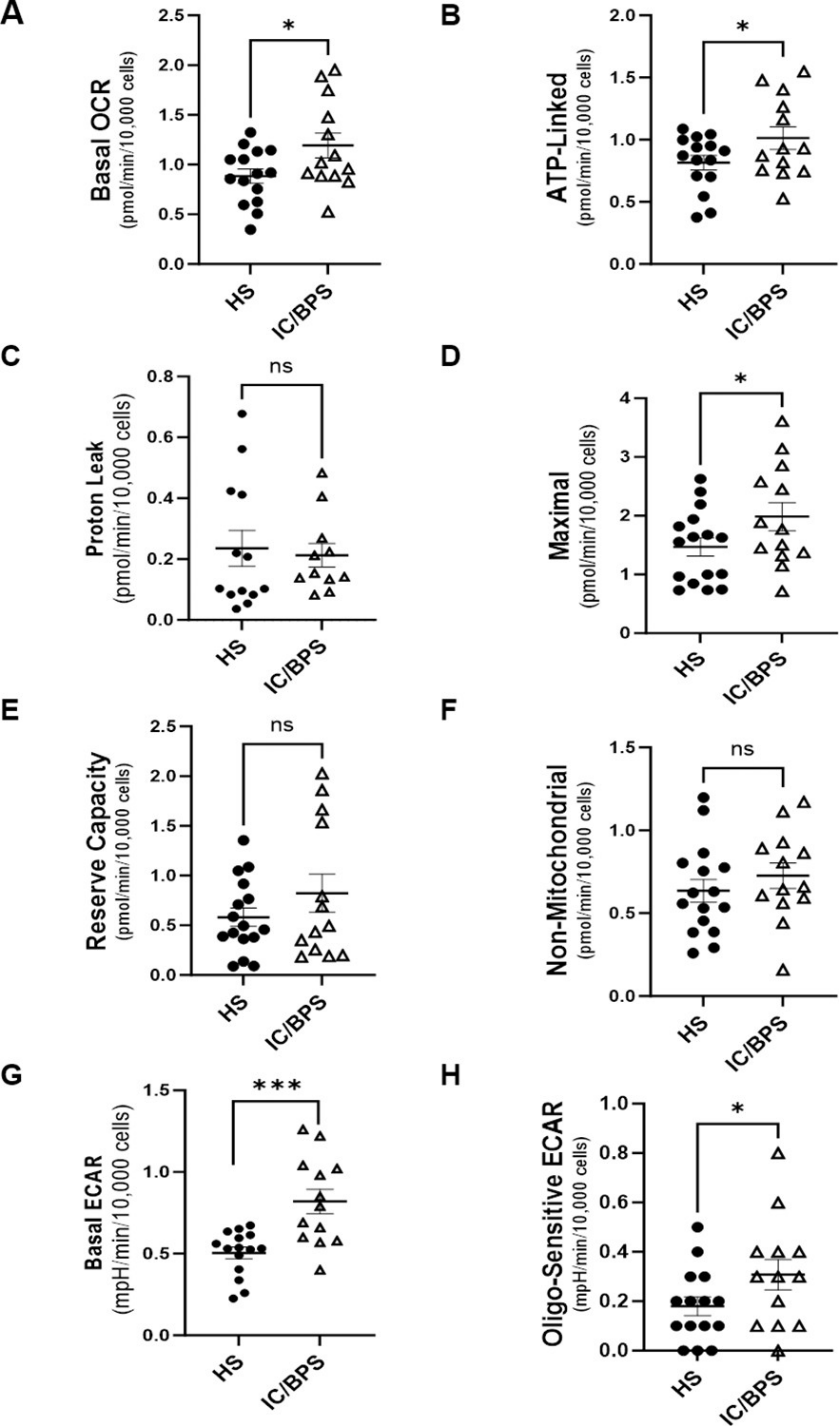
Lymphocyte mitochondrial respiration and glycolysis in healthy subjects and patients with Interstitial Cystitis/Bladder Pain Syndrome (IC/BPS). Distribution of (A) basal, (B) ATP-linked, (C) Proton Leak, (D) Maximal, (E) Reserve Capacity, and (F) Non-mitochondrial OCR parameters as well as (G) Basal ECAR and (H) Oligo-sensitive ECAR parameters in lymphocytes from study participants. Data expressed as mean ± SD, n = 5–6 replicates; up to n  = 16 HS, n = 13 IC/BPS. *p<0.05, ***p<0.001 different from HS.

**Fig 2** shows the distribution of basal, ATP-linked, proton leak, maximal, reserve capacity, and non-mitochondrial respiration in lymphocytes from HS and patients with IC/BPS. Basal (0.88 ± 0.28 vs 1.19 ± 0.44 pmol/min/10,000 cells; p = 0.018, **Fig 2A**), ATP-linked (0.82 ± 0.23 vs 1.01 ± 0.33 pmol/min/10,000 cells; p = 0.035, **Fig 2B**), and maximal respiration (1.47 ± 0.63 vs 1.99 ± 0.87 pmol/min/10,000 cells; p = 0.037, **Fig 2D**) were significantly higher in lymphocytes from patients with IC/BPS than HS. However, proton leak (0.24 ± 0.21 vs 0.21 ± 0.13 pmol/min/10,000 cells; p = 0.379, **Fig 2C**), reserve capacity (0.58 ± 0.37 vs 0.82 ± 0.69 pmol/min/10,000 cells; p = 0.121, **Fig 2E**), and non-mitochondrial OCR (0.64 ± 0.27 vs 0.73 ± 0.28, **Fig 2F**) were not significantly different between the two groups. In addition, both basal ECAR (0.50 ± 0.18 vs 0.82 ± 0.27 mpH/min/10,000 cells; p = 0.001) (**Fig 2G**) and Oligo-sensitive ECAR (0.18 ± 0.15 vs 0.31 ± 0.22 mpH/min/10,000 cells; p = 0.040) (**Fig 2H**) were significantly higher in patients with IC/BPS compared to healthy subjects.

### Plasma cytokine levels

**Fig 3:**
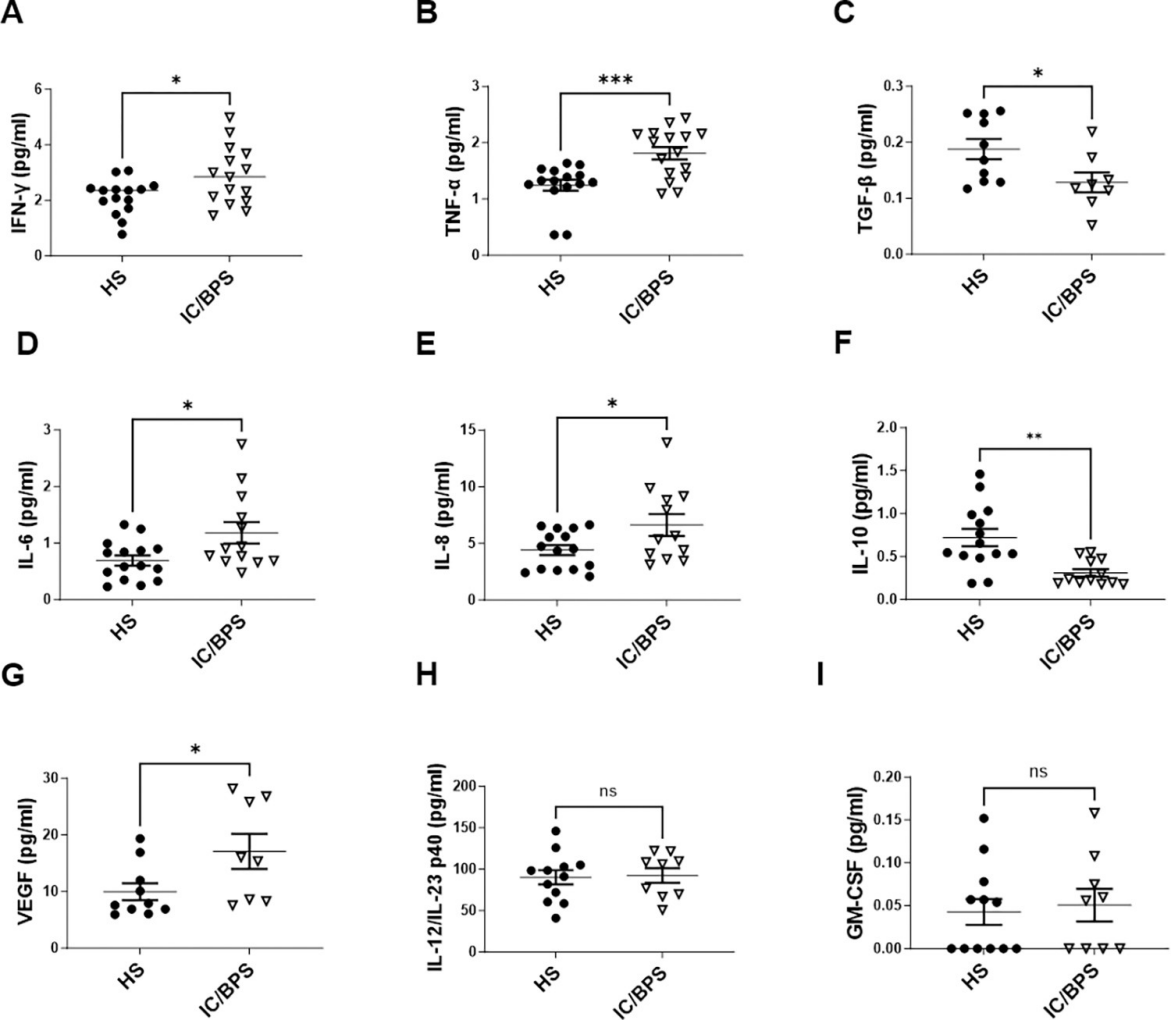
Plasma pro-inflammatory cytokine levels in healthy subjects (HS) and patients with Interstitial Cystitis/Bladder Pain Syndrome (IC/BPS). Distribution of plasma (A) IFN-ɣ (B) TNF-ɑ, (C) TGF-β, (D) IL-6, (E) IL-8, (F) IL-10, (G) VEGF, (H) IL-23/23 p40, and (I) GM-CSF levels in study participants; Results are presented as mean ± SD; up to n  = 15 HS, n = 16 IC/BPS *p<0.05 compared to HS.

Several cytokine and chemokine analytes were measured in the plasma of both cohorts using MSD Technology (**Fig 3**). IFN-ɣ, TNF-ɑ, TGF-β, IL-6, IL-8, IL-10, and VEGF plasma levels were significantly different in patients with IC/BPS compared to HS (**Fig 3**). IFN-ɣ is a cytokine secreted by the innate and adaptive immune systems and is known to activate macrophages [21]. IFN-ɣ plasma levels were significantly elevated in patients with IC/BPS compared to HS (2.12 ± 0.63 vs 2.88 ± 1.05 pg/ml; p = 0.029, **Fig 3A**). Plasma TNF-ɑ levels were also significantly elevated in patients with IC/BPS compared to HS (1.25 ± 0.38 vs 1.81 ± 0.44 pg/ml; p = 0.001, **Fig 3B**). TNF-ɑ is mainly secreted by macrophages and is involved in regulating a number of cellular processes such as cell proliferation and cell death [22]. In contrast, plasma TGF-β levels were significantly reduced in the individuals with IC/BPS (0.19 ± 0.06 vs 0.13 ± 0.06 pg/ml; p = 0.034, **Fig 3C**). TGF-β is an important regulatory cytokine for maintenance of immune homeostasis by controlling the proliferation, differentiation and survival of lymphocytes [23]. Other pro-inflammatory cytokines, IL-6 and IL-8, were also significantly upregulated (0.69 ± 0.35 vs 1.18 ± 0.68 pg/ml (p = 0.022) and 4.42 ± 1.70 vs 6.62 ± 3.33 pg/ml (p = 0.035) respectively, **Fig 3D and 3E**); whereas, IL-10, an anti-inflammatory cytokine, was reduced in patients with IC/BPS compared to healthy subjects (0.72 ± 0.38 vs 0.31 ± 0.15 pg/ml; p = 0.002, **Fig 3F**). Vascular endothelial growth factor (VEGF) is another pro-inflammatory cytokine that was elevated in the plasma of individuals with IC/BPS (9.99 ± 4.72 vs 17.09 ± 8.76 pg/ml; p = 0.042, **Fig 3G**). VEGF plays an important role in vascularization and the infiltration of monocytes and macrophages to inflammatory sites [24]. Lastly, IL-12/IL-23p40 and GM-CSF were not significantly different between the two groups (90.27 ± 29.58 vs 92.54 ± 26.44 pg/ml (p = 0.858) and 0.043 ± 0.052 vs 0.051± 0.056 pg/ml (p = 0.743), respectively, **Fig 3H and 3I**).

### Correlation coefficients

**Fig 4:**
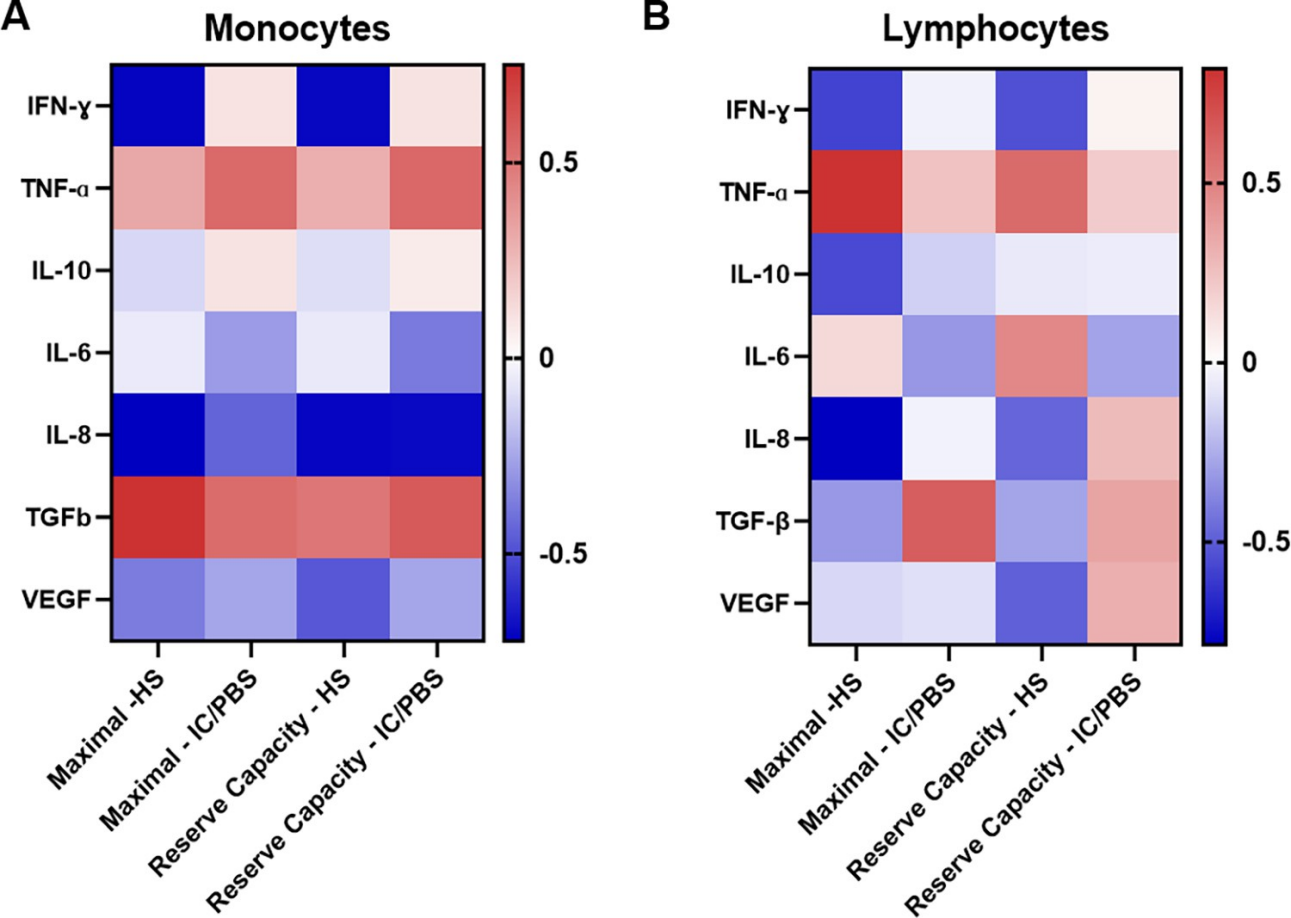
Correlation heat map between mitochondrial metabolic responses and cytokine levels in monocytes and lymphocytes from healthy subjects (HS) and patients with Interstitial Cystitis/Bladder Pain Syndrome (IC/BPS). The parametric correlation coefficients of (A) monocyte or (B) lymphocyte maximal respiration/reserve capacity oxygen consumption rates vs various plasma cytokine levels are shown. Red shades represent positive correlations and blue shades represent negative correlations. Data are from n = 18 HS and n = 18 IC/BPS.

To determine whether plasma cytokine values correlated with monocyte and lymphocyte mitochondrial parameters, Pearson correlation coefficients were determined. The unadjusted correlation coefficients of mitochondrial maximal and reserve capacity OCR with plasma cytokines for both monocytes and lymphocytes are shown in **Fig 4 and S1 Table**. IFN-ɣ was inversely associated with maximal OCR (R = -0.7132, p = 0.021) and reserve capacity (R = -0.7041, p = 0.023) in monocytes from HS. IFN-ɣ did not have a significant relationship with either mitochondrial parameter in IC/PBS patients. IL-8 also had an inverse correlation with both maximal respiration (R = -0.723; p = 0.043) and reserve capacity (R = -0.709, p = 0.048) in monocytes from HS, while IL-8 was inversely correlated only with reserve capacity (R = -0.699, p = 0.049) in monocytes from IC/PBS patients. TGF-β was positively correlated with maximal respiration (R = 0.748, p = 0.042) in HS monocyte only. In lymphocytes, TNF-ɑ was positively correlated with maximal OCR (R = 0.818, p = 0.024) in HS, whereas IL-8 was inversely correlated (R = -0.787, p = 0.02) in HS. No significant associations were observed with any of the studied cytokines in lymphocytes from IC/PBS patients.

## Discussion

Cellular metabolism plays an important role in regulating immune cell function. Disruption of mitochondrial respiration and glycolysis in immune cells by inflammation and oxidative stress during a systemic disease can disrupt the immune response. In this study, we investigated whether cellular bioenergetics in monocytes and lymphocytes is different in women with IC/BPS compared to healthy women and its association with inflammatory cytokines/chemokines. We observed a disruption in cellular bioenergetics in monocytes and lymphocytes in IC/BPS patients. In addition, the levels of inflammatory cytokines (IFN-γ, TNF-α, IL-6, IL-8) were higher in the plasma whereas the levels of regulatory cytokines (TGF-β and IL-10) were reduced in those with IC/BPS patients. We also observed correlations between plasma TNF-ɑ and IL-8 levels and monocyte mitochondrial respiration. These findings suggest a role of circulating monocytes and lymphocytes in the pathogenesis of IC/BPS.

It has been shown in several studies that immune cell bioenergetics is altered in systemic diseases [13–18]. The ability of these cells to function properly is vital for cellular homeostasis and tissue repair [12, 25]. Consistent with this, monocyte mitochondrial respiration was decreased in our IC/BPS cohort. During activation, lymphocytes can increase both mitochondrial respiration and glycolysis [26]. The mitochondrial properties of lymphocytes in IC/BPS patients were also energetically different than HS. Lymphocytes from IC/BPS had elevated mitochondrial respiration and glycolytic rates which suggests they may be activated and more energetic than lymphocytes from HS, which could be important for modulating an immune response. In addition, it appears lymphocytes are more active in IC/BPS compared to monocytes, which suggests that adaptive immunity may be a major source of immunity in this cohort.

It is understood that cytokines and chemokines may be involved in IC/BPS [1, 27]. A few groups have assessed whether evaluating these analytes in the urine and serum of patients could serve as potential biomarkers of the condition [8, 28, 29]. We examined a number of cytokines/chemokines in the plasma from our study cohorts. Of the analytes measured, plasma IFN-ɣ, TNF-ɑ, IL-6, Il-8 and VEGF cytokine levels were the only analytes to be significantly elevated in IC/BPS patients. IFN-ɣ is known to activate macrophages and to upregulate other pro-inflammatory cytokines such as TNF-ɑ, IL-12, and IL-15 [21]. TNF-ɑ has been shown to stimulate mast cell activation and increase urothelial inflammation through activation of nuclear transcription factor-kB (NF-kB) signaling [30]. Our findings are similar with previous work where Jiang et al. determined that TNF-ɑ is elevated in IC/BPS patients [8]. Exposure of monocytes to IFN-ɣ or TNF-ɑ can lead to the production of inflammatory M1 macrophages [12]. Thus, such cytokine/chemokine signaling could alter cellular bioenergetics and thereby dysregulate the immune response. Other studies have also reported increased inflammatory cytokine levels (IL-6 and IL-8) in IC/BPS [31, 32]. Further, IL-6 has been established as a sensitive and specific biomarker for IC/BPS [33]. In addition, IL-8 levels are higher in patients with ulcerative vs non-ulcerative IC/BPS suggesting it may be a potential marker for ulcerative IC/BPS [28, 34]. It has also been reported that an increase in VEGF levels correlates with the increased inflammation, angiogenesis, and bladder pain in IC/BPS patients [35, 36]. We also determined that the regulatory cytokines (TGF-β and IL-10), which are thought to play important role in tissue homeostasis, were significantly reduced in this cohort which may contribute to increased tissue damage in patients [35]. Collectively, these findings corroborate previous studies suggesting that inflammation is elevated in IC/BPS patients.

Another major finding from this study was identifying an association of cytokines with cellular bioenergetic parameters in monocytes and lymphocytes. In particular, the mitochondrial parameters, maximal respiration and reserve capacity, were the only parameters associated with elevated TNF-ɑ cytokine levels in monocytes. These two parameters are indicators of mitochondrial health and the cell’s ability to respond to stress. The correlation of elevated TNF-ɑ levels with decreased mitochondrial maximal and reserve capacity in monocytes suggests that the energetic capacity in monocytes is affected by increased inflammation. Additionally, IL-8 displayed an inverse correlation with reserve capacity. Although these findings highlight a potential relationship between the mitochondrial parameters, still further studies are warranted with a larger sample size to establish correlations between plasma cytokine levels and the cellular bioenergetics of immune cells.

The idea of evaluating cellular bioenergetics in circulating immune cells to assess disease progression or to monitor the benefit of treatment in pathologies has been suggested [20, 26, 37, 38] and could be applied to this patient population in the future based on our findings. We acknowledge that the present study has limitations. Samples were collected from some patients with a history of additional comorbidities (i.e. hypertension, hypothyroidism, fibromyalgia or endometriosis). In addition, standard cellular and acellular blood markers were not evaluated and blood collection occurred once prior to pain treatment management. It would have been insightful to determine the pain score at the time of the blood draw as well as the performance of quantitative sensory testing and whether these correlated to inflammatory or mitochondrial responses. Assessing the effects of pain management on immune cell function and plasma pro-inflammatory cytokine levels was also not done and will be considered for future studies. Another limitation of the study was that only women were enrolled. Determining whether these responses occur in men with IC/BPS warrants further investigation and could be useful to understand any potential sex differences. Nevertheless, this study provides insight regarding the bioenergetic profile of immune cells in IC/BPS and suggests that evaluating changes in metabolism in peripheral immune cells may be useful to differentiate IC/BPS phenotypes.

## Conclusions

Monocyte and lymphocyte metabolism is altered to varying degrees in women with IC/BPS compared to healthy women. The results of this study suggest that lymphocytes may be more bioenergetically active than monocytes in IC/BPS patients. In addition, TNF-ɑ plasma levels correlated positively with both maximal respiration and reserve capacity parameters in monocytes in IC/BPS. In contrast, IL-8 plasma levels correlated negatively with reserve capacity in monocytes from patients with IC/BPS. No statistically significant correlations were observed in lymphocytes in the IC/BPS group compared to HS. These data suggest that inflammation and metabolism in monocytes and lymphocytes could be important in understanding IC/BPS pathogenesis. To our knowledge, this is the first study to report these associations. Additional studies are needed to evaluate a larger cohort of patients and to understand the cross talk between inflammatory cytokines and immune cells from patients with IC/BPS. Findings from more planned studies could be useful to help understand IC/BPS epidemiology and guide therapeutic treatment in this cohort.

## Supporting information

S1 Table Parametric correlation coefficients of cytokine levels and mitochondrial responses in monocytes and lymphocytes. (PPTX)

S1 Data (XLSX)
