# Supplementary material for: Bioenergetic profiles of peripheral mononuclear cells and systemic inflammation in women with Interstitial Cystitis/Bladder Pain Syndrome (IC/BPS)
Source: PLoS One. 2024 Feb 15;19(2):e0298981. doi: 10.1371/journal.pone.0298981 (PMC10868762; doi:10.1371/journal.pone.0298981)
Supplement: S1 Table — (PPTX) [file pone.0298981.s001.pptx]

## Slide 1
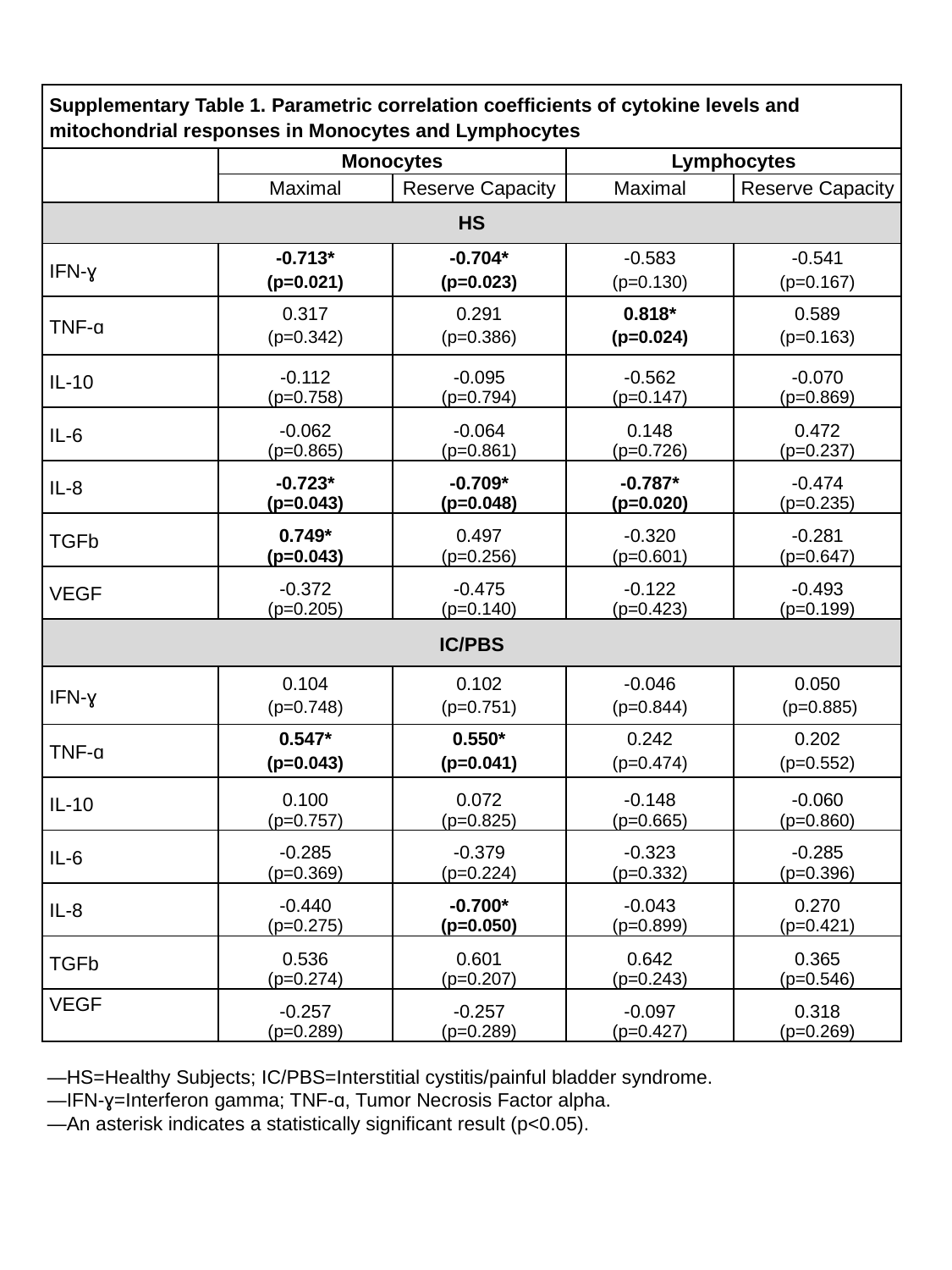

| Supplementary Table 1. Parametric correlation coefficients of cytokine levels and mitochondrial responses in Monocytes and Lymphocytes | | | | |
| --- | --- | --- | --- | --- |
| | Monocytes | | Lymphocytes | |
| | Maximal | Reserve Capacity | Maximal | Reserve Capacity |
| HS | | | | |
| IFN-ɣ | -0.713\* (p=0.021) | -0.704\* (p=0.023) | -0.583 (p=0.130) | -0.541 (p=0.167) |
| TNF-ɑ | 0.317 (p=0.342) | 0.291 (p=0.386) | 0.818\* (p=0.024) | 0.589 (p=0.163) |
| IL-10 | -0.112 (p=0.758) | -0.095 (p=0.794) | -0.562 (p=0.147) | -0.070 (p=0.869) |
| IL-6 | -0.062 (p=0.865) | -0.064 (p=0.861) | 0.148 (p=0.726) | 0.472 (p=0.237) |
| IL-8 | -0.723\* (p=0.043) | -0.709\* (p=0.048) | -0.787\* (p=0.020) | -0.474 (p=0.235) |
| TGFb | 0.749\* (p=0.043) | 0.497 (p=0.256) | -0.320 (p=0.601) | -0.281 (p=0.647) |
| VEGF | -0.372 (p=0.205) | -0.475 (p=0.140) | -0.122 (p=0.423) | -0.493 (p=0.199) |
| IC/PBS | | | | |
| IFN-ɣ | 0.104 (p=0.748) | 0.102 (p=0.751) | -0.046 (p=0.844) | 0.050 (p=0.885) |
| TNF-ɑ | 0.547\* (p=0.043) | 0.550\* (p=0.041) | 0.242 (p=0.474) | 0.202 (p=0.552) |
| IL-10 | 0.100 (p=0.757) | 0.072 (p=0.825) | -0.148 (p=0.665) | -0.060 (p=0.860) |
| IL-6 | -0.285 (p=0.369) | -0.379 (p=0.224) | -0.323 (p=0.332) | -0.285 (p=0.396) |
| IL-8 | -0.440 (p=0.275) | -0.700\* (p=0.050) | -0.043 (p=0.899) | 0.270 (p=0.421) |
| TGFb | 0.536 (p=0.274) | 0.601 (p=0.207) | 0.642 (p=0.243) | 0.365 (p=0.546) |
| VEGF | -0.257 (p=0.289) | -0.257 (p=0.289) | -0.097 (p=0.427) | 0.318 (p=0.269) |
—HS=Healthy Subjects; IC/PBS=Interstitial cystitis/painful bladder syndrome.
—IFN-ɣ=Interferon gamma; TNF-ɑ, Tumor Necrosis Factor alpha.
—An asterisk indicates a statistically significant result (p<0.05).
